# Assessing the Impact of Recommendation Novelty on Older Consumers: Older Does Not Always Mean the Avoidance of Innovative Products

**DOI:** 10.3390/bs14060473

**Published:** 2024-06-05

**Authors:** Li Zhao, Bing Fu

**Affiliations:** College of Business Administration, Capital University of Economics and Business, Beijing 100070, China; lizhao666@hotmail.com

**Keywords:** personalized recommendations, recommendation novelty, cognitive age, stereotype threat, receptiveness to innovativeness

## Abstract

Personalized recommendations that use digital technologies to predict user interests and preferences and give guiding conclusions have become a widely used digital marketing tool on e-commerce platforms. Given that existing consumer behavior research has not reached a consensus on the relationship between age and the adoption of innovative products, whether recommendation novelty can stimulate older consumers’ acceptance of innovative products remains uncertain. Grounded in the aging and social influence literature, this experimental study investigated the moderating role of individual cognitive age on the impact of recommendation novelty on consumer perceptions regarding stereotype threat and receptiveness to innovativeness. An experiment involving 239 online shoppers was conducted to investigate the experiences of cognitively younger and older adults while using low or high levels of recommendation novelty designed for this study. Results reveal the tension for older adults when using highly recommended novelty, as they perceive these to be more of a stereotype threat, but they also have a higher level of receptiveness to innovativeness. This finding is contrary to the common belief that “the older the consumer, the less receptive to innovativeness”, providing novel insight into the information systems literature. Theoretically, this research shows how increasing the level of recommended novelty affects stereotype threat and receptiveness to innovativeness (of consumers of different cognitive ages). For practitioners, the results provide important guidelines on the kind of personalized recommendations that are appropriate for consumers with different cognitive ages.

## 1. Introduction

Personalized recommendations that assist consumers in examining, evaluating, and comparing products in the context of their preferences by analyzing information from consumers’ history on e-commerce platforms [1,2], such as implicit feedback (e.g., dwell time, clicks) and explicit feedback (e.g., reviews, purchases), and provide them with online purchase recommendations that match their interests [3], which play a critical role in helping consumers to reduce product information overload and improve the quality of their online purchasing decisions, have become a commonly used digital marketing tool on e-commerce platforms [4,5,6,7]. The accuracy of predicting consumer needs and preferences is a fundamental principle in personalized recommendation research [8]. To evaluate the performance of personalized recommendation systems, most of the existing studies focus on “recommendation accuracy” [9,10,11]. However, accuracy alone is not sufficient to adequately reflect users’ potential interests [12]. Consumers’ preferences and needs are unstable during the process of shopping for commodities [13], and they not only need relatively accurate information about commodity recommendations but may also need information about commodities that are novel, unfamiliar, and relevant [14]. Novelty metrics have attracted increasing attention in the field of recommender systems, and recommending unseen or unexpected items to consumers, especially the more popular items that are novel to them, is an optimization strategy to cope with the unpredictability of consumer preferences and provide a set of different items to capture consumers’ potential interests [14].

Recommendation novelty promotes innovative product presentation and diffusion, and the acceptance of novel recommendations implies a tendency to purchase new and different products or brands rather than repeating previous choices and consumption patterns [15]. This concept of consumer innovativeness is often considered in marketing practice because it is directly related to the success of new product sales and promotion [16]. However, common knowledge and some empirical studies suggest that innovation is considered to be the dominant characteristic of younger consumers [17], with older consumers preferring older brands and traditional products [18,19]. Nevertheless, with the intensifying growth of the aging population and the depth of digitalization, the Internet is penetrating the daily lives of older adults in all aspects and having a great impact on their lifestyles [20]. While older adults have a strong interest in online shopping [21], they cannot avoid being influenced by personalized recommendations from e-commerce platforms. Older adults have become an important population for information systems researchers and online retailers to consider. However, due to physical and cognitive limitations associated with the natural aging process, people tend to hold negative stereotypes of older adults as having diminished physical abilities or capabilities [22]. These negative age-based perceptions predispose older consumers to feel stereotype threatened and to perceive themselves as being treated with prejudice and discrimination [23], and they will attempt to defend such negative evaluations by withdrawing from trying new things [24,25]. Several studies have demonstrated that this stereotype-threatening self-assessment causes older consumers to avoid innovations [26,27]. However, it has also been suggested that the relationship between age and innovativeness adoption is not simply linear [28] and that there may be no difference in the propensity to adopt innovations between younger and older consumers [29]. Stereotype confrontation theory and self-protection mechanisms also point to the fact that when confronted with age-related stereotype-threatening factual statements or cues, older consumers move away in ways that are expected to be the opposite of their age group and engage in compensatory consumption behaviors that are manifested in liking innovative products [30,31]. It is evident that consumer behavior research has failed to reach a consensus on the relationship between age and innovative product adoption. Currently, empirical evidence testing the applicability of personalized recommendations for novelty to older age groups is lacking.

Research suggests that cognitive limitations are not absolute with age [32]. Individually relevant socioemotional and meaningful information can motivate older adults to deploy cognitive resources flexibly, allowing them to perform comparably to younger adults [33]. It has also been suggested that the lack of valid new information for older adults due to the loss of social connections is a factor in older adults’ limited decision making [34]. Social influence theory states that social influence may drive an individual’s choice process, helping them to make more carefully considered and less delayed purchases [35]. Due to cognitive impairments associated with aging, the cognitive limitations of older adults often lead them to rely more heavily on social norms and others’ opinions for their decisions and behaviors. Social influence thus plays a crucial role in the elderly population. However, the loss of work and family connections among older adults may reduce the social influence they receive, leaving those who receive fewer recommendations unaware of alternative products in the market, thereby lacking the motivation to purchase new or substitute products [35]. This may partly explain why older consumers avoid new choices and purchase from a familiar, smaller consideration set. Social influence refers to changes in individuals’ beliefs and behaviors due to their social relationships, manifesting as older consumers adapting to others’ purchase decision opinions [36]. When older adults receive brand or product purchase advice information comparable to that of younger adults, this may drive them to think more carefully about purchase options and delay purchases less, thereby directly influencing purchase decisions. Thus, although based on the ideas commonly presented in the literature on aging and innovative use [19,25,27] it may be assumed that “the older an individual is, the less receptive they are to innovativeness”, depending on the information-driven profile of social influence, this study suggests that recommendation novelty as a form of social influence, while creating a higher perceived stereotype threat to older consumers, may also facilitate the process of acceptance of innovative products by older consumers and assist them in making more appropriate decisions.

The majority of existing studies dealing with personalized recommendations and their design have focused mainly on the efficiency of recommendation algorithms, younger age groups, and gender, with a lack of understanding of what happens in the use of personalized recommendations due to age differences, as well as a lack of exploring the impact on the outcomes of older age groups in terms of novelty indicators that are closely related to their characteristics [37,38,39]. In addition, it has been shown that the occurrence of aging varies from one individual to the other, that actual age does not accurately reflect changes in an individual’s physical systems and cognitive abilities [40], and that it is cognitive age that is a better predictor of an individual’s perceptions and behaviors [1]. Therefore, this study utilizes the theory of aging and social influence, uses cognitive age to measure younger and older age groups, and empirically examines the tension that exists between cognitively older adults in the use of novel product recommendations using a situational experiment questionnaire. This study attempts to resolve this “unknown” in the information systems (IS) literature on novelty and older age group research in order to assess the interrelationships between the novelty of personalized recommendations for older adults’ understanding of their own aging and consumer receptiveness to innovativeness. The empirical findings of this study provide important guidelines for online marketers recommending new products and brands, which should be considered when designing new product recommendations for different age groups. 

## 2. Theoretical Background

### 2.1. Recommendation Novelty

Novelty refers to the difference between present and past experiences and is defined in terms of the number of users with specific knowledge of an item, frequently indicated by adjectives such as unknown, unexpected, surprising, and unfamiliar [41]. Recommendation novelty typically involves recommending a product or service that is relevant to consumers’ needs and preferences but is different from what has been “previously seen” or experienced, and it can be considered as the extent to which an unseen item is different from a known item [42]. The recommended product or service is novel if it is new, unknown, or unfamiliar and relevant to the consumer [43]. Novel recommendations avoid redundancy, enhance users’ range of choices, help resolve uncertainty in user preference prediction, help users discover new things motivate them to explore potential areas of interest, and enrich the user experience over time [44]. In this study, we define recommendation novelty as a personalized recommender system that recommends new and more popular products and services that are relevant to consumers’ needs and different from their previous knowledge and experience based on their historical purchase history. Recommendations with a low degree of novelty only provide consumers with products and services that are highly relevant to their past experiences, and the recommended items do not contain any new information for consumers. On the other hand, recommendations with a higher degree of novelty require that consumers be offered new products or services that are unfamiliar or popular to a greater degree than their expected purchase experience.

### 2.2. Aging and Innovative Product Adoption

According to the theory of aging, older adults suffer from limitations in their cognitive abilities [45], and they begin to experience a steady decline in the speed at which they process information, their working memory, and their long-term memory [46]. Cognitive deficits associated with aging may mean that older adults have difficulty remembering and processing information, especially new information. This impairment may lead to a simplification of the consumer choice process and a tendency to select well-known options. The decline of physical factors, such as worse vision or difficulty in walking, may limit their mobility and thus exacerbate the simplification of the choice process. As a result, older people are commonly perceived as facing negative stereotypes of illness, unattractiveness, and cognitive decline [47]. These negative perceptions make older consumers vulnerable to age-based stereotype threats [31], prompting older consumers to abstain from trying new products and services [24,27]. The reasons why stereotype threat causes older consumers to avoid the use of innovative products may be reflected in the following areas.

First, risk aversion can lead older adults to choose to avoid innovative products and draw them to more familiar options that may be less risky [35]. A study of risk aversion found that older consumers were more likely to express risk-related concerns [48]. Older people’s goals will reflect more loss avoidance and maintenance orientations than younger and middle-aged people [32]. In the face of negative stereotype threats that cause tension and anxiety [49], older adults respond to identity threats by withdrawing their efforts and/or moving away from target domains that discriminate against them [27]. Older consumers who want to maintain their “ageless” age identity decide to avoid unfamiliar products to counteract age-perception threats [25,50]. That is, older consumers threatened by negative age stereotypes are reluctant to choose new brands or products [24].

Another explanation for older consumers’ avoidance of innovative product use is socioemotional selectivity associated with risk aversion. Socioemotional selectivity theory states that older adults tend to be attentive to emotional information as they age [51]. When older adults perceive that the time left in the future is very limited, they prioritize staying with more familiar social partners who can provide trustworthy emotional rewards [52]. This leads older consumers to focus more on established emotional ties than on acquiring new information, which may result in a preference for long-term known choices, exhibiting stronger loyalty to pre-existing choices and lower rates of trying new products [53,54].

Consistent with socioemotional selectivity are nostalgia, attachment, and habitat mechanisms that may also contribute to older consumers’ preference for traditional products [53]. Nostalgia is a preference for objects (or people) that were more common (popular, fashionable, or widely circulated) in one’s youth [55]. According to nostalgia theory, consumers develop preferences during “critical periods” of youth, such as between the ages of 15 and 30, and maintain them throughout their lives [56]. The attachment mechanism suggests that consumers develop an emotional connection to frequently used items or brands over time because they are psychologically identified with their use [57]. In addition, a habitat mechanism also suggests that habits become stronger with age and that older adults may be more inclined to choose options that have been used for a long time [32]. Thus, based on the existing literature, it is widely believed that the older consumers are, the more they prefer established options and avoid innovative product use. Given this general view, in this study, we wanted to explore whether this is also true in the case of novel personalized recommendations in online shopping.

### 2.3. Social Influence Theory

Previous research on decision making in older adults suggests that conservatism among older buyers may be related to cognitive decline, with older adults shifting to repeated choices or postponing purchases if the cognitive effort involved in their decision-making process is increased [53]. However, there is also evidence that speed rather than ability to process information is measured in tests of cognitive processes and that older adults’ more extensive knowledge may offset their cognitive deficits [58]. This suggests that older adults may have the ability to make effective decisions, but they may seldom utilize this ability because of their slower processing speed and may make more thorough and quicker decisions when stimulated by social influences with more information and advice. Thus, social influence plays a role in the purchase choice decisions of older consumers.

Social influence has been defined as changes in an individual’s beliefs and behaviors as a result of their social relationships and environment [59]. The strength of social influence has been shown for a long time in bandwagon effect research [60], and East et al.’s study states that social influence may drive the choice process of individuals, helping them to make more carefully considered and less delayed purchases. Social influence depends on social contact, which in turn depends on the individual’s connection to society and group membership based on factors such as education, neighborhood, work, friendship, and family [35]. Old age often implies limited physical activity or intellectual decline [61], which is usually accompanied by a gradual loss of social connections such as work and family and greater dependence on others [62,63]. This reduction in work and social connections results in older consumers being less influenced by their social relationships and environments than younger consumers and may have difficulty receiving advice on purchase decisions from others [34]. Older consumers may be more interested in certain product categories if they receive a comparable amount of advice to younger adults in those areas [35]. As a means of social influence in online environments, recommendation novelty presents newly emerging, popular, and recently launched products or services to older consumers in the form of purchase recommendations, increasing their acceptance of the new product or service.

The influence of the social environment on consumer behavior in general is well demonstrated both in practice and empirically [64,65]. When older adults encounter younger social cues in their consumption environment, older consumers’ age-related identities may become more visible in their minds, and negative aging stereotypes may become more sensitive [31]. Because the presence of younger groups represented by younger cues may prompt older adults to make intergroup comparisons [66], they may become more aware of not being young enough to pose an age-related threat [23]. Prior research on stereotype threat suggests that this threat may induce older adults to adopt disengagement strategies to distance themselves from their age group as a way of self-protecting themselves from the threat of negative stereotypes of aging [67]. In addition, individuals also have engagement strategies to cope with stereotype threat, whereby, when faced with explicit factual statements or cues of age-related performance decline, participants sometimes behave in ways that are contrary to the expectations of stereotypical perceptions, to dispel the negative stereotypes that others have of them [26]. Amatulli et al.’s study confirmed that youthful social cues trigger aging-related identity threats in older consumers in immediate environments, yet they generate compensatory consumption behaviors and provide evidence that they prefer contemporary products to traditional ones when they feel young [31]. Based on these findings, we argue that older consumers who receive novel personalized recommendations of rejuvenation cues stimulate stereotypical perceptions of aging, but that they perceive themselves as more receptive to new recommended products or services when considering self-protective mechanisms, especially under the influence of access to new information that can be used for decision making [68]. Therefore, older consumers who are faced with the influence of novel recommendations will be more likely to accept the recommended product or service, showing a higher level of receptiveness to innovativeness, because it provides more comprehensive information about the purchase recommendation, and they can achieve the goal of moving away from the threat of stereotypes by accepting that type of product. 

### 2.4. Cognitive Age

Chronological age (number of years since birth) is one of the most commonly used demographic variables in marketing research and has been widely used as a key descriptive statistic and grouping variable in segmentation and targeting [69]. Furthermore, aging research has shown that most adults tend to feel younger than their chronological age and this tendency becomes more pronounced as they age [31]. Therefore, age is showing that it is more of a mental state than a physical state [70], and an individual’s perception of aging should be measured against their self-awareness of aging [40]. As a result, some studies have used an individual’s subjective perception of aging as a measure of age [71]. Amatulli et al. pointed out that in many cases, age in chronological order may not be sufficient to understand consumer behavior, especially for older age groups [31]. Some evidence also suggests that the purchasing behavior of older consumers is better explained by their perceived age rather than their chronological age [72]. This cognitive age, which individuals derive from their self-perceptions of how they look, feel, and behave, and the interests they have, has been the most commonly used age measure in consumer research [1]. 

The results of the research in this field also suggest that cognitive age is a more accurate predictor of people’s beliefs, attitudes, and behaviors than age because it more accurately reflects this nonconstant rate of aging [73]. Individuals with younger cognitive ages report higher self-efficacy compared with older individuals [74]. For several older adults, the difference between their chronological age and their perceived age creates new identities and behaviors [75] that can express some of the service needs that differentiate them from their chronological age group through a relatively younger identity [71]. Several studies confirm that consumers’ perceived age affects consumption decisions. For example, older people who feel younger prefer contemporary products to traditional counterparts [31] and are more likely to try new brands and products [54]. Established research has also confirmed that older consumers with younger cognitive ages have a positive impact on innovativeness and are more willing to test new things and exhibit innovative behaviors [24,76].

Meanwhile, Hong et al. found that individuals’ cognitive age is a better predictor of their perceptions and behaviors toward using online technologies than their chronological age [77]. In addition, most consumers on online platforms that do not mandate real names usually provide falsified age data, weakening the reliability of the measure in terms of chronological age. Moreover, cognitive age correlates well with chronological age and is a better predictor of cognitive decline and related attitudinal changes [78]. Therefore, in this study, we use cognitive age as a more appropriate measure of age to understand how age affects consumers’ responses in the face of novel personalized recommendations. This is because, as mentioned above, many previous studies have argued that it is a better predictor of an individual’s attitudes and behaviors. It reflects individuals’ assessment of their cognitive abilities, which in turn influences their perceptions of stereotype threat and receptiveness to innovativeness in this study.

**Figure 1:**
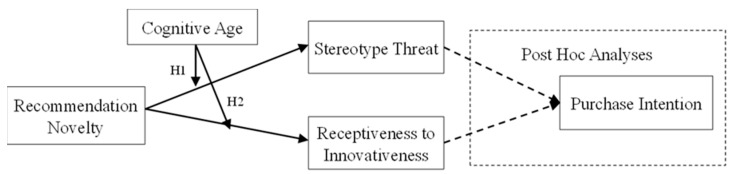
Research Model.

Based on the above analysis, this study constructs a theoretical model that illustrates the interaction effects between consumer cognitive age and recommendation novelty on perceived stereotype threat and receptiveness to innovativeness, grounded in theories of aging and social influence, as shown in Figure 1. Since in marketing and consumer behavior research, understanding the impact of different psychological factors on purchase intention is essential to demonstrate practical business value, considering the influence of perceived stereotype threat and receptiveness to innovativeness on purchase intention across different cognitive age groups can further reveal the practical implications of recommendation novelty in these groups. Therefore, in the research model depicted in Figure 1, we included a post hoc analysis of the impact of perceived stereotype threat and receptiveness to innovativeness on consumers’ purchase intention.

## 3. Hypotheses Development

### 3.1. Stereotypes Threaten Perception

Stereotype threat is the perceived threat of judging an individual’s behavior based on negative stereotypes [66]. The view on stereotype threat suggests that negative self-related group stereotypes cause a person to experience stereotype threat and situational anxiety because people think of themselves based on these negative stereotypes [66]. Younger people are allegorized to be more active, healthy, free, strong, and innovative [79], and aging is often associated with a decline in cognitive and physical functioning. These negative stereotypes expose older people to a generalized threat of negative stereotypes that is different from that of younger people [73]. This threat can be viewed as a social identity threat that is perceived when older adults perceive to encounter situational cues that suggest marginalization of their group [23]. For older consumers, this may include exposure to new technologies and novelties associated with rejuvenation such as social media, online shopping, and smart technologies [80,81]. In this study, we argue that novel recommendations that represent young people’s identities trigger stereotype threat in older adults.

Research shows that an individual’s cognitive age is an important predictor of their cognitive ability, with younger cognitive age predicting better cognitive functioning [82]. In general, cognitively younger adults have better mental and physical functioning [83], and they can be psychologically closer to the younger people around them by expressing a younger identity [84]. By expressing a youthful identity, older adults protect themselves from the threats associated with the negative aspects of aging [27]. As individuals age, people anticipate their aging process and older adults perceive more anxiety about identity threats if they are strongly aware of their aging [85]. Several studies have shown that cognitively older adults perceive higher levels of threat of physical decline than cognitively younger adults [67]. Additionally, it has been noted that those who perceive themselves to be younger than their chronological age are more optimistic about their ability to maintain memory and other aspects of cognition [86]. Thus, cognitively older adults are expected to experience higher perceived stereotype threats when presented with a novel product or service recommendation compared with cognitively younger adults. Accordingly, we hypothesized the following:

**H1.**  *Cognitive age moderates the effect of recommendation novelty on increasing perceived stereotype threat, such that the effect is stronger in cognitively older adults.*

### 3.2. Consumer Receptiveness to Innovativeness

Innovativeness is related to novelty seeking and has been described as an internal drive or incentive to seek novel information that is being activated by the individual, often thought of as a preference for new perceptions and different sensory experiences [87]. Consumer innovativeness is defined as the tendency to adopt and accept new and different products or brands [15]. Highly innovative consumers are more likely to explore multiple sources of information and embrace new consumer experiences, whereas less innovative consumers prefer familiar choices [16,88]. Therefore, consumer innovativeness as a potential consumer psychological trait for accepting new products is often seen as an important factor in the influence of innovative product adoption intentions. In this study, we investigated the moderating role of cognitive age in the effect of recommendation novelty on consumer receptiveness to innovativeness. 

There are two contradictory strands of reasoning regarding the effect of cognitive age on recommended novelty on receptiveness to innovativeness. First, several studies have shown that aging hurts innovativeness, that innovation is often perceived as a trait possessed by younger age groups [17], and that older adults prefer to make choices about familiar products or traditional brands [85]. Because older consumers respond to the threat of aging stereotypes posed by novelty by avoiding unfamiliar products or services [22], this avoidance affects older consumers’ receptiveness to innovativeness [50]. On the other hand, cognitive age is an important predictor of an individual’s cognitive ability and is a more conscious understanding of the behavioral-based aspects of individual aging [40]. Older adults who feel younger can be more likely to try new brands and purchase new products by expressing a youthful identity [31]. Recommendation novelty brings new product or service information, increases new consumer perceptions and experiences, and increases stereotype threat perceptions among cognitively older adults. These novel recommendations decrease the level of receptiveness to innovativeness of cognitively older adults, as they may have difficulty accepting these recommended products or services compared with cognitively younger adults.

Nevertheless, individuals’ participatory strategies for coping with stereotype threat suggest that older adults’ cognitive deficits are manifested as reduced processing speed rather than diminished ability, that their ample knowledge and experience may offset cognitive deficits [89], and that it is the loss of social connections that is the primary factor in older adults’ limited appraisal of what is going on [34]. Reduced social connectedness means that older adults do not have the same easy access to information for decision-making as younger adults, leading to a common choice of avoidance strategies to deal with stereotype threats. The “human–computer interaction” created by personalized recommendation systems that meet predictions of individual preferences and interests compensates for older adults’ reduced social connections due to retirement and physical limitations. In addition, as a social context that influences older adults’ consumption in online shopping, recommendation novelty prepares older adults for potentially more comprehensive and popular product recommendations that are relevant to their interests and preferences, thus providing new information for purchase decisions, and they will adopt an engaged purchasing strategies in response to the threat of stereotyping. As predicted by social influence theory, older adults will be more interested in these novel products when they receive a comparable amount of advice as younger adults on in-demand product information [65]. From this perspective, while recommendation novelty leads to a higher level of conscious stereotype threat for cognitively older adults, they are also more likely to perceive themselves as having a higher level of receptiveness to innovativeness compared with cognitively younger adults. Because cognitively younger adults have higher levels of innovativeness themselves, they do not perceive additional innovativeness from novel recommendations. Thus, on the balance of the above arguments, we hypothesize the following:

**H2.**  *Cognitive age moderates the effect of recommendation novelty on increasing consumer receptiveness to innovativeness, such that the effect is greater for cognitively older adults.*

## 4. Research Methodology

### 4.1. Experimental Design

To distinguish between novel recommendations, in this study, we designed an empirical investigation of recommendation novelty using an experimental approach of helping participants choose a door lock. For most older adults who spend a great deal of their time at home, more money and energy are spent on building their own better home environments; so, home goods are well suited to be experimented with in this study. The product of door locks was chosen for the focus of this study because their technology is constantly being updated, old and new technology can be applied to different scenarios and needs, the degree of novelty can be distinguished well, and they are a household product that both young and old people may buy.

We selected two categories of products, traditional mechanical door locks and electronic smart door locks, based on product data from Taobao (www.taobao.com), China’s largest online shopping platform, to form the basis for designing the different recommended novelties in this experimental study. To minimize the impact of confusion due to branding or design elements, both recommended novelties focus on different brands and design styles. Considering the actual situation of simulating the mobile Taobao App, which displays four products per screen and consumers swipe the screen to browse, the designed personalized recommended products were identified as eight out of twelve products in different categories by a group discussion of five Ph.D. students, as shown in Table 1. Different categories of personalized recommended products show different degrees of novelty, according to two groups of door lock product categories using two different web page parameters to simulate the design of the Taobao online shopping recommended display interface (see Appendix A). The participants, by clicking on the set parameter links, can enter the display interface. In each category, the product display image can be clicked on to enter the Taobao platform’s detail page interface, where participants can browse and evaluate the product’s parameters in the detail page interface. We expect that participants will have significantly different perceptions of the novelty of personalized recommendations in the low and high treatment groups shown in Table 1.

### 4.2. Measurements of Variables

To ensure content validity, previously validated instruments were used to measure constructs in this study. Recommendation novelty was coded as a dummy variable (i.e., 1 for low novelty and 2 for high novelty). Cognitive age was measured using a mean based on Barak and Schiffman’s median values for the four age dimensions [90]. Ghasemaghaei et al. also used these items in a study of recommended agents to measure older adults, with an internal consistency reliability of 0.98 points [1]. Perceived stereotype threat was measured using Bae et al.’s three-item scale, with the scale slightly adapted to reflect the context of this study [24]. Receptiveness to innovativeness was adapted from Baumgartner and Steenkamp’s Exploratory Purchasing Scale (EXP) [91] and referenced Roehrich’s Adaptive Innovation Scale to finalize the four question items [92]. They were measured using a Likert-type five-point scale (1 = strongly disagree, 5 = strongly agree). In this study, six potentially relevant control variables were also included in the survey, including participants’ interest in purchasing door locks, participants’ general knowledge of door locks, online shopping experience, gender, education level, and income level. All control variables were measured with individual question items. All measurement items are included in Table 2. The scales for purchase intention are also included in Table 2, as the effects of stereotype threat perception and consumer innovativeness on purchase intention will be examined later in the Section 5.4.

### 4.3. Sample

Samples were randomly selected from a sample of ordinary online shoppers from China. To ensure the number of participants in both young and old age groups, we purposefully controlled the number of each age group during the sample selection process. At the same time, to motivate participants to take the experimental task seriously, some participants were given the possibility of receiving a cash prize of RMB 2–10 upon completion of the experiment. Participants were randomly assigned via the web to one of two experimental personalized recommendation treatments (i.e., low or high recommendation novelty) and were asked to complete a door lock purchase task using their assigned recommended item. They were then asked to click on a web link for survey completion at the bottom of the merchandise display page to answer perceived age questions and complete the survey. To ensure that we had enough cognitively younger and cognitively older adults in our sample, we took a snowball approach of 500 participants through a web-based experiment. Of these 500 participants, 463 provided usable data, 51.8% were female, 72.4% had a college degree or higher, and 61.1% had an average monthly income of less than RMB 5000. The statistical distribution of cognitive age relative to actual age is shown in Appendix B.

To support our analysis of the moderating role of cognitive age on the relationship between recommendation novelty and stereotype threat perception (H1) and between recommendation novelty and receptiveness to innovativeness (H2), we calculated participants’ cognitive age based on their responses to questions on the cognitive age scale. For the analysis in this study, we focused on comparing two specific cognitive age groups. The first group consisted of cognitively younger adults (20 < cognitive age ≤ 30), and the second group consisted of cognitively older adults (cognitive age > 60). These two cognitive age ranges were chosen for analysis because individuals belonging to these two groups in chronological age have significantly different cognitive abilities, with the younger group having significantly higher cognitive abilities than the older group [94]. These differences are expected to remain when comparing these groups based on cognitive age, as the older cohort with a higher biological age still has a much higher cognitive age than the younger group [77]. Based on the responses of our 463 participants to the cognitive age scale, 141 participants were ultimately categorized as cognitively younger adults, while 98 were categorized as cognitively older adults.

### 4.4. Statistical Analysis

Given that this study employs a 2 × 2 factorial design, we utilized Analysis of Variance (ANOVA) to examine the moderating effect of cognitive age on the relationships between recommendation novelty and stereotype threat (H1), as well as recommendation novelty and consumer innovativeness (H2). The ANOVA allows for the comparison of means across multiple groups to determine if there are significant differences between them. The ANOVA can handle single-factor situations, and through a Multivariate Analysis of Variance (MANOVA), it can simultaneously analyze multiple factors and their interactions with the dependent variable. Therefore, in this study, we used SPSS 23.0 statistical software to conduct both a multivariate and a univariate ANOVA to test our research hypotheses.

Since Partial Least Squares Structural Equation Modeling (PLS-SEM) can analyze measurement and structural models with multiple items, including multigroup comparisons, we selected PLS-SEM for the post hoc analysis of purchase intention. Using Smart PLS 4.0, we constructed structural equation models and performed a multigroup analysis (Bootstrap Multigroup Analysis) to examine the impact of perceived stereotype threat and consumer innovativeness on consumers’ purchase intention for novel recommended products in online shopping.

## 5. Data Analysis and Results

### 5.1. Manipulation Check

After the experimental task was completed (using either low recommendation novelty or high recommendation novelty), each participant was asked to answer the following two questions on a five-point Likert scale (very low to very high).

(1) Please indicate your perception of the type and number of recommended door locks as an overall difference from past merchandise experiences.

(2) Please indicate your perception that the recommended door lock merchandise stimulated potential interest in the door locks you were looking for.

For the first question, participants who used low recommendation novelty reported a mean of 2.41 (standard deviation of 0.87), whereas participants who used high recommendation novelty reported a mean of 3.77 (standard deviation of 0.89). This difference was significant (one-way ANOVA, *p* < 0.001). For the second question, participants who used low recommendation novelty reported a mean of 2.53 (standard deviation of 0.81) on the manipulation check question, whereas participants who used high recommendation novelty reported a mean of 3.69 (standard deviation of 0.87). This difference was also significant (one-way ANOVA, *p* < 0.001). Hence, our manipulation of recommendation novelty was successful.

### 5.2. Construct Validation

To evaluate the reliability of the measurement items, the loadings of each measurement item on its intended construct were assessed and compared with the recommended tolerance of 0.70. As Table 3 shows, each measurement item had a loading value greater than 0.7 on its intended construct and also was an order of magnitude larger than any other loading. 

As can be seen in Table 4, to demonstrate the internal consistency of the constructs, the composite reliability and Cronbach’s alpha were calculated for each construct across our sample of cognitively younger and older adults (*n* = 239). As shown in Table 4, the Cronbach’s alpha and composite reliability scores for all the constructs were above 0.7, indicating good reliability of the measures. Meanwhile, the average extracted variance (AVE) values of each measurement item were greater than 0.5, demonstrating satisfactory convergent validity [95]. Additionally, the correlation coefficients between each construct (Table 4) were lower than the square root of the AVE (diagonal value), indicating good discriminant validity [95]. Table 5 shows the means and standard deviations of the variables.

### 5.3. Tests of the Moderation Hypotheses

To test the moderating effect of cognitive age on the relationship between recommendation novelty and stereotype threat (H1), as well as recommendation novelty and consumer receptiveness to innovativeness (H2), ANOVAs were conducted to understand whether any differences exist between cognitively older and younger adults’ perceptions following a 2 × 2 factorial design (i.e., low cognitive age (between 20 and 30)/high cognitive age (above 60) × low recommendation novelty/high recommendation novelty). First, we conducted a multivariate analysis of variance (MANOVA) on both perceived stereotype threat and consumer innovativeness together. Pillai’s trace tests revealed a significant main effect of recommendation novelty (*p* < 0.001), as well as a significant interaction effect between recommendation novelty and cognitive age (*p* < 0.001). Then, we conducted follow-up ANOVAs to test the effects on stereotype threat and consumer receptiveness to innovativeness separately. The ANOVA measures also yielded significant effects of recommended novelty and the interaction term between recommended novelty and age of cognition on perceived stereotype threat, as well as consumer receptiveness to innovativeness (see Table 6 and Table 7). 

**Figure 2:**
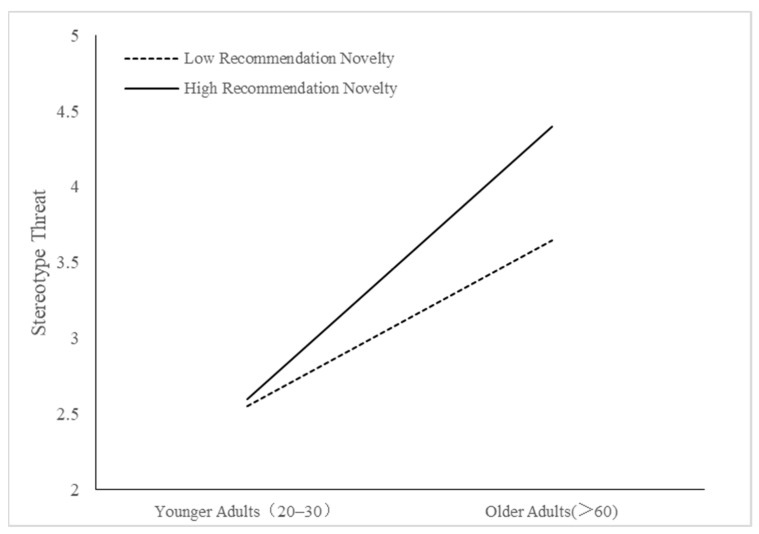
Interaction effect between cognitive age and novelty level of recommendation on perceived stereotype threat.

**Figure 3:**
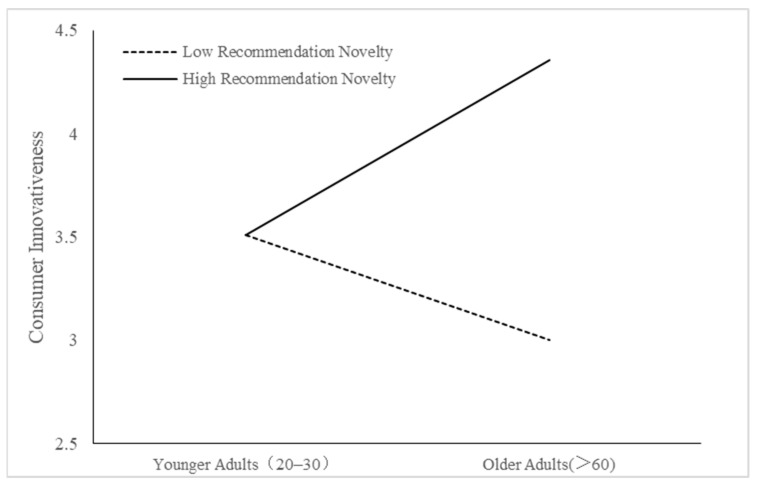
Interaction effect between cognitive age and novelty level of recommendation on consumer receptiveness to innovativeness.

We also conducted multiple comparative analyses to assess whether individuals’ perceptions of stereotype threat and consumer innovativeness differed when cognitively older and younger adults used lower or higher levels of recommended novelty. The results of these analyses are displayed in Figure 2 and Figure 3. As hypothesized, cognitive age was found to moderate the relationship between recommendation novelty and stereotype threat perceptions, resulting in a stronger effect on cognitively older adults. Thus, H1 was supported. Also, cognitive age was found to moderate the relationship between recommendation novelty and consumer receptiveness to innovativeness, which was stronger for cognitively older adults. Therefore, H2 was also supported. Recommendation novelty had no significant effect on stereotype threat and receptiveness to innovativeness for cognitively younger adults. On the other hand, cognitively older adults perceived high levels of recommendation novelty to be perceived as higher levels of stereotype threat and promoted high levels of receptiveness to innovativeness. Finally, results also show that the control variables did not significantly influence the dependent variables in our study.

### 5.4. Post Hoc Analyses

We used Smart PLS 4.0 to construct structural equation modeling for Bootstrap Multigroup analysis to examine the effects of stereotype threat and receptiveness to innovativeness on consumers’ purchase intention of novel recommended products in future online shopping. We found that the difference in the perceived impact of stereotype threat on purchase intention between the cognitively older and younger adult groups was not significant (Δβ = −0.049, *p* = 0.629). The difference in the perceived impact of receptiveness to innovativeness on purchase intention between the cognitively older and younger adult groups was highly significant (Δβ = 0.518, *p* < 0.001). This differential relationship becomes significant when only the effect of stereotype threat perception on purchase intention in the absence of receptiveness to innovativeness is considered (∆β = 0.338, *p* < 0.01). This also illustrates the key role played by receptiveness to innovativeness in the acceptance and purchase of their innovative products in the cognitively older adult group and further demonstrates the practical effect of recommendation novelty on different cognitive age groups.

## 6. Contributions, Limitations, and Future Research

### 6.1. Theoretical Contributions

This study contributes to the aging consumer literature by analyzing how different levels of recommended novelty (low or high) affect stereotype threat and receptiveness to innovativeness perceptions among consumers of different cognitive ages. First, this study aims to identify the reasons for the retention of innovation awareness among older consumers from a social influence perspective, and the findings of this study contribute to our understanding of the interrelationships between age consciousness and the understanding of one’s own aging and receptiveness to innovativeness. Existing recommender system research has focused on young people or recommender system design [1,96], and there is a lack of research on age acceptance of recommended novelty goods. We explain how consumers’ cognitive age moderates the impact of recommended novelty on their perception of stereotype threat and receptiveness to innovativeness when shopping online by applying aging and social influence theories. In this study, we identify the tension that cognitively older adults have when confronted with novel product recommendations, as they perceive them as bringing stereotype threat and are more open to accepting and purchasing them. We advance the personalized recommendation literature by considering the impact of users’ cognitive age on the design of novelty goods in personalized recommendations, which constitutes a novel finding on how changes in social information in online environments affect the consumption of innovative products by older consumers.

As expected, cognitive age was found to moderate the relationship between recommendation novelty and perceived stereotype threat. This suggests that older adults who perceive themselves as older may have their perceptions of the stereotype threat of aging deepened by novel merchandise recommendation messages that represent rejuvenation. This may be because an individual’s cognitive age is an important predictor of their cognitive ability, with cognitively younger adults predicting better cognitive functioning [82] and greater optimism about their cognitive ability to retain memories and other aspects of their cognitive abilities [86], which affects their social group connections and motivation for external information acquisition, and in turn, decreases their perception of stereotypes in recommending novel products. Contrary to the common belief in the literature that the older the individual is, the less innovative the products they accept [24], cognitive age has been found to moderate the relationship between recommended novelty and consumer receptiveness to innovativeness: it has a stronger effect on cognitively older adults. Due to limitations in social connectedness and access to social information, cognitively older adults have a stronger need for novel offers; thus, the marketing environment of personalized recommendations generates higher perceptions of receptiveness to innovativeness. Although previous research has shown that younger cognitive age has a positive impact on receptiveness to innovativeness [76], older adults threatened by negative age stereotypes avoid choosing new brands or products [27]. However, these explain the process of new product adoption by older consumers from the perspective of avoidance and lack the argument that they generate compensatory consumption behaviors from the perspective of engagement strategies. Our results suggest that older adults, whose cognitive deficits are manifested as reduced processing speed rather than diminished capacity [58], under the influence of access to more comprehensive and popular suggestions of new product information that can be used in decision making, choose to adopt a purchasing strategy that moves them away from the stereotypes threat. Thus, cognitively older adult consumers find that recommended high novelty triggers higher levels of stereotype threat perception than low-novelty goods, but this stereotype threat perception may have stimulated their intention to explore new things, considering that they believe that this stereotype threat perception adds to the absence of information about trendy, more popular product recommendations in their current social connections.

Subsequently, different from most information system studies that focus on the actual age of individuals, this study tested the important role of cognitive age in the impact of recommendation novelty by using the cognitive age of individuals to support their ability to be a better predictor of receptiveness to innovativeness than their chronological age. In addition, the results of previous research suggest that cognitive age is a better age criterion for measuring consumer behavior toward personalized recommendations [77]. Therefore, counter to most online marketing studies that focus on the role of age in Internet technology adoption, in this study, we use individuals’ cognitive age rather than just their actual age. Our ANOVA results are also consistent with Ghasemaghaei et al.’s results on cognitive age being a more appropriate age criterion for measuring consumer perceptions of recommendation comprehensiveness affecting recommender agent complexity and usefulness [1], further validating that cognitive age results are more consistent with theories of aging and supporting their validity for predicting older consumers’ perceptions of stereotype threat and online products’ receptiveness to innovativeness. These findings are consistent with neurological evidence suggesting that cognitive limitations are not absolute with age [32] and that cognitive limitations in the acceptance of novelty can be improved depending on the social context and informational influences.

### 6.2. Practical Contributions

This study also has important implications for practitioners interested in targeting older consumers. Older consumers are an important market segment not only because they are expanding the consumer population but also because they have more household assets and more disposable income than younger consumers [97]. These realities underscore the need to deepen our understanding of how older consumers feel and behave in different consumption environments. The results of this study reveal that both cognitively older and younger consumers in personalized recommendation environments perceive recommended high-novelty items as having high levels of receptiveness to innovativeness compared with low-novelty items. In particular, the finding that cognitively older adults perceive recommended high-novelty items as contributing more to their level of receptiveness to innovativeness provides new insight. Our study demonstrates that older adults respond to recommendations as well as younger adults. Such results suggest that marketing communications can be as successful among older adults as among other groups. Since our post hoc analysis shows that consumer receptiveness to innovativeness is the most significant factor influencing users to purchase the recommended novel items, our recommendation to personalized recommendation service providers is that they should consider offering some new item options for all age groups, as opposed to habitual or well-known item options that should be recommended for older adults, as well as items that can be relevant to their purchasing needs. They should also provide more comprehensive products that are relevant to their purchasing needs and provide more comprehensive information. Although older adults may continue to avoid adopting innovations if their characteristics and needs are not well understood [98], novelty recommendations can help them find products that are relevant to their needs and complementary to their interests and preferences and can guide new products and brands in their pursuit of overcoming this barrier to older people’s access to detailed product information. This is particularly important for cognitively older adults, who have limited social connections and access to information due to biological and physical factors and can make better choices using the wealth of knowledge and life experience they already have when given adequate product information and recommendations in the marketplace. Reconsidering older consumers’ perceptions of aging and innovation will help to increase the adoption rate of innovative products. Moreover, they may show more loyalty to the novel products they purchase due to habitual and socioemotional factors and provide repeat business and positive word-of-mouth advertising to their online suppliers.

### 6.3. Limitations and Future Research

Although this study provides substantial insights into theory and practice, it has several limitations. First, the questionnaire data collected for our study were from China, which may limit the generalizability of this study. Consumers living in Eastern cultures (e.g., China and Japan) are more collectivist than Western cultures (e.g., the U.S. and the U.K.) [99], and older adults’ roles in society and perceptions of receptiveness to innovativeness may differ in more collectivist cultures. Further research is needed to generalize and validate the findings of this study using a diverse set of participants who are older adults. Second, this study was conducted with participants in a personalized recommendation environment using a simulated real e-commerce platform. But this approach only selected one product and was differentiating between items with different novelty. In a real shopping environment, products with different degrees of novelty and in different categories are displayed in the same recommendation list. Consumers may not have entirely consistent feelings about the mixed presentation of personalized recommendation lists. Therefore, further evaluation is needed to assess the perceived level of stereotype threat and receptiveness to innovativeness from the simultaneous presentation of items with varying degrees of novelty, and whether the effects of these factors vary by age. Finally, in this study, we implicitly captured the effects of physical and cognitive decline by measuring the cognitive age of individuals. However, it is possible that participants could have influenced their cognitive age through the marketing stimuli of contextual social cues [31]. Next research could explore measuring individuals’ actual physical and cognitive limitations through appropriate cognitive tests to objectively determine their abilities and as a way to understand their responses to recommended novelty items, rather than using cognitive age to represent these abilities.

Future research can be conducted in several directions. First, previous research has shown that the mental state of “flow” is a characteristic of online environments, and thus affective factors can influence user experience and shopping decisions in e-commerce environments [100]. Although affective factors were not the focus of this study, future research could explore how these factors influence the experience of consumers of different cognitive ages with different recommended novelty items. Second, the results of this study suggest that while recommended high-novelty items increase stereotype threat perceptions among older adults in the context of online shopping decisions, they also increase the level of innovativeness among cognitively older consumers. Future research should investigate whether the results of this study can be generalized to different categories of personalized recommendation systems or to shopping platforms that focus on innovative product recommendations. Furthermore, future research could use other types of online decision support tools (e.g., online reviews) to examine associations with our present study in other promotional contexts (e.g., social media) [101]. Finally, the task of purchasing a door lock is simple and common, and consumers may find that recommended items with high novelty promote receptiveness to innovativeness perceptions more than items with low novelty because they can easily judge and accept new items in that category. Therefore, in tasks where innovation complexity is high and difficult to assess (e.g., purchasing an automobile), consumers may find that the overall performance of novelty is not significant because the core functionality of the automobile does not make a focused breakthrough for them, and they do not need to make additional adaptations to make their own purchasing decisions. Thus, future research could assess whether individuals’ perceptions differ when they are engaged in shopping tasks with higher levels of complexity.

## 7. Conclusions

This study addresses an important gap in the information systems literature in understanding the impact of age on recommendation novelty in consumers’ perception of stereotype threat and receptiveness to innovativeness, a novel aspect of information systems research that has not been previously considered. Using individual perceived age as a measure of age, this study reveals how consumers’ cognitive age affects their perceptions of using personalized recommendations for novelties. This study also explores the tension that exists when older consumers use high levels of recommendation novelty, as they perceive higher levels of stereotype threat perceptions from recommendation novelty, but it also promotes levels of receptiveness to innovativeness in older consumers. This finding provides new insight into the information systems literature, as it is contrary to mainstream recommendations for older consumers who are typically provided with traditional, well-known choices for purchases. Considering that the elderly population is rapidly increasing, that they are the fastest-growing segment of Internet users, that they show a strong interest in online shopping, and that they have become a fast-growing group of information technology users in most countries, these results are an important insight into the implications of older consumers for business practices.

## Figures and Tables

**Table 1:** Design of Recommendation Novelty.

	Low Recommended Novelty	Highly Recommended Novelty
Product Categories	Traditional mechanical door locks	Electronic smart door lock
Number of products	8	8

**Table 2:** Measurement Items.

Constructs	Measures	Sources
Cognitive Age (COA)	COA1: I feel as though I am my age.	Barak and Schiffman (1981) [90]
	COA2: I look as though I am my age.	
	COA3: I do most things as though I were my age.	
	COA4: My interests are mostly those of a person of my age	
Stereotype Threat(STT)	STT1: Sometimes I feel that other people treat me as an older adult.	Bae, Jo, and Lee (2021) [24]
	STT2: Some people feel that I am not good at work because of my age.	
	STT3: Some people feel that I have poor memory because of my age.	
Receptiveness to Innovativeness (RTI)	RTI1: When I see a new or different product, I often want to see what it is like.	Baumgartner and Steenkamp (1996) [91]; Roehrich (2004) [92]
	RTI2: When I see a new or different product for sale, I am not afraid of giving it a try.	
	RTI3: A new store or restaurant is something I would be eager to find out about.	
	RTI4: I am more interested in buying new than known products.	
Purchase Intention (PUI)	PUI1: It is likely that I will consider buying the gate lock with recommendations if I need it.	Yi, Jiang, and Benbasat (2015) [93]
	PUI2: I may purchase the recommended goods the next time I need them.	
	PUI3: Supposing a friend contacts me for advice on buying a gate lock, I would recommend the goods that are recommended online.	

**Table 3:** Loading and Cross-Loading of Measure.

Constructs	STT	RTI	PUI
Stereotype Threat (STT1)	**0.902**	0.207	0.274
Stereotype Threat (STT2)	**0.899**	0.162	0.232
Stereotype Threat (STT3)	**0.921**	0.158	0.235
Receptiveness to Innovativeness (RTI1)	0.092	**0.780**	0.503
Receptiveness to Innovativeness (RTI2)	0.219	**0.784**	0.478
Receptiveness to Innovativeness (RTI3)	0.105	**0.763**	0.451
Receptiveness to Innovativeness (RTI4)	0.177	**0.753**	0.464
Purchase Intention (PUI1)	0.134	0.534	**0.891**
Purchase Intention (PUI2)	0.276	0.559	**0.913**
Purchase Intention (PUI3)	0.308	0.537	**0.845**

Note: As cognitive age and recommendation novelty are single-item measures that result in loadings of 1.000, they were not included in this analysis. Bold in numbers in the table indicates the load of each measurement item on its intended construct.

**Table 4:** Internal Consistency and Discriminant Validity of Constructs.

	CA	CR	AVE	REN	COA	STT	RTI	PUI
Recommendation Novelty (REN)	-	-	-	**1**				
Cognitive Age (COA)	0.992	0.992	0.977	0.069	**0.989**			
Stereotype Threat (STT)	0.894	0.904	0.824	0.176	0.676	**0.908**		
Receptiveness to Innovativeness (RTI)	0.771	0.774	0.593	0.358	−0.050	0.195	**0.770**	
Purchase Intention (PUI)	0.859	0.860	0.781	0.362	−0.025	0.274	0.616	**0.884**

Note: CA = Cronbach’s alpha; composite reliability = CR; diagonal elements bold number are the square root of AVE; off-diagonal elements are correlations among constructs.

**Table 5:** Descriptive Statistics with means and Standard Deviations (SD).

Groups	Stereotype Threat		Receptiveness to Innovativeness		Purchase Intention	
	Mean	SD	Mean	SD	Mean	SD
Low (n = 121)	2.983	1.042	3.370	0.657	3.265	0.742
High (n = 118)	3.381	1.194	3.837	0.569	3.808	0.660
Total Sample (n = 239)	3.180	1.135	3.600	0.657	3.533	0.752

Note: Low: low recommendation novelty; High: high recommendation novelty.

**Table 6:** ANOVA Summary Table for Stereotype Threat.

Source	df	Mean Square	F	Sig.
Intercept	1	100.273	176.096	0.000
Gender	1	4.925	8.649	0.004
Education	1	0.401	0.703	0.403
Income	1	8.643	15.163	0.000
Online Shopping Experience	1	0.024	0.042	0.838
Purchase Interest	1	3.587	6.299	0.013
Product Knowledge	1	0.410	0.721	0.397
Recommendation Novelty	1	8.435	14.813	0.000
Cognitive Age	1	48.251	84.737	0.000
Recommendation Novelty × Cognitive Age	1	6.625	11.634	0.001
Error	229	0.569		
Total	239			

**Table 7:** ANOVA Summary Table for Consumer Receptiveness to Innovativeness.

Source	df	Mean Square	F	Sig.
Intercept	1	50.015	192.984	0.000
Gender	1	0.520	2.007	0.158
Education	1	0.034	0.130	0.719
Income	1	0.051	0.199	0.656
Online Shopping Experience	1	2.035	7.853	0.006
Purchase Interest	1	1.131	4.366	0.038
Product Knowledge	1	1.816	7.007	0.009
Recommendation Novelty	1	24.510	94.572	0.000
Cognitive Age	1	0.675	2.606	0.108
Recommendation Novelty × Cognitive Age	1	23.040	97.048	0.000
Error	229	0.259		
Total	239			

**Figure A1:**
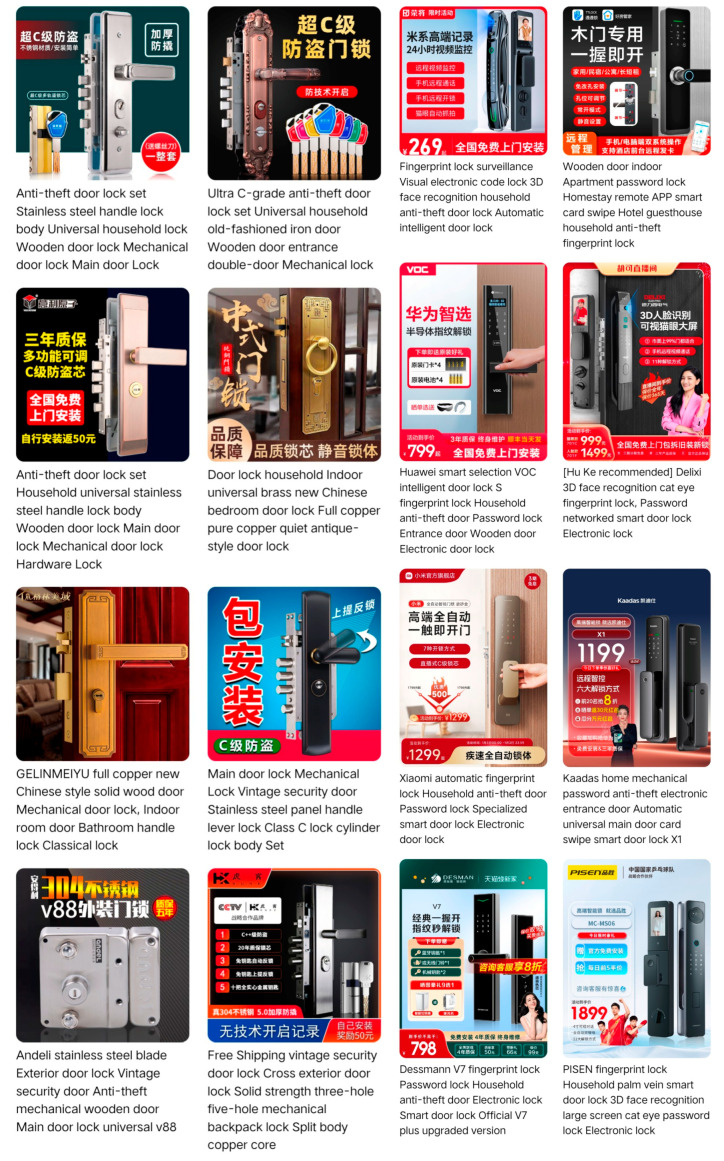
Low and high recommendation novelty in the data gathering stage.

**Figure A2:**
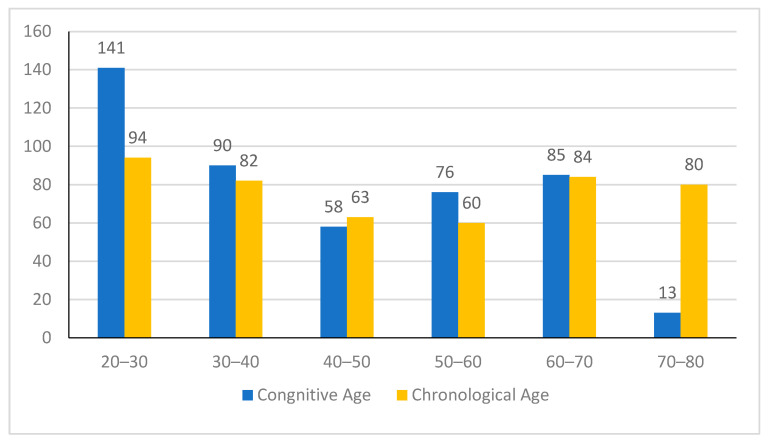
Distribution of participants’ cognitive and chronological age.

## Data Availability

The data are available from the authors upon reasonable request.

## Appendix B. Group Comparisons between Cognitive Versus Chronological Age

**Table A1:** Chronological age versus cognitive age.

	Cognitive Age						
	20–30	30–40	40–50	50–60	60–70	70–80	Total
20–30	84	7	3	0	0	0	94
30–40	44	32	6	0	0	0	82
40–50	8	39	11	5	0	0	63
50–60	5	9	28	18	0	0	60
60–70	0	3	10	48	20	3	84
70–80	0	0	0	5	65	10	80
Total	141	90	58	76	85	13	463
